# Localized RAS signaling drives cancer

**DOI:** 10.18632/oncoscience.479

**Published:** 2019-04-01

**Authors:** Sandip K. Basu, Srikanta Basu, Peter F. Johnson

**Affiliations:** Mouse Cancer Genetics Program, Center for Cancer Research, National Cancer Institute, Frederick, MD, 21702 USA

**Keywords:** cancer, RAS, perinuclear signaling complexes (PSCs), signaling endosomes, senescence

Dysregulated signaling through the core RAS pathway (RAS-RAF-MEK-ERK) provides the oncogenic drive for many cancers. Activating mutations in RAS GTPases or RAF kinases occur in approximately 40% of human malignancies, and many others contain mutant or overexpressed receptor tyrosine kinases (RTKs) that induce aberrant signaling through the RAS pathway. *RAS* cancers are particularly aggressive and are refractory to current therapeutic interventions (mainly RAF and MEK inhibitors), typically due to narrow therapeutic windows, paradoxical pathway activation, feedback induction of PI3 kinase/Akt signaling, and/or drug resistance [1]. Moreover, RAS proteins are not particularly amenable to direct inhibition by small molecules. Therefore, to identify new therapeutic targets for *RAS* cancers it is imperative to further elucidate the biochemistry and cell biology of RAS effector pathways, with the goal of identifying actionable differences between normal and oncogenic RAS signaling.

Mutant *RAS* or *RAF* oncoproteins generally induce high signaling output in cancer cells (i.e., elevated p-ERK); however, this is not always the case. For example, a comprehensive molecular characterization of human lung adenocarcinomas revealed that p-ERK levels remain moderate or low in a substantial proportion of tumor samples, including many that harbor *KRAS* mutations [2]. One interpretation of these findings is that high RAS-ERK output is not necessarily required for tumorigenesis, suggesting that qualitative features of RAS signaling may also play a role.

We have uncovered novel aspects of RAS signaling by investigating oncogenic RAS-induced post-translational activation of the transcription factor, C/EBPβ. C/EBPβ is an auto-inhibited protein that undergoes multiple activating modifications upon oncogenic RAS signaling. In primary cells such as MEFs, activated C/EBPβ is a key effector of oncogene-induced senescence (OIS) and cell cycle arrest [3,4]. In addition, C/EBPβ regulates transcription of senescence-associated secretory phenotype (SASP) genes, which include inflammatory cytokines, their receptors, secreted metalloproteases and other factors. Unexpectedly, we previously found that C/EBPβ activation in tumor cells is suppressed by sequences in the 3’ untranslated region (3’UTR) of its mRNA [5]. This form of regulation, termed “3’UTR regulation of protein activity” or UPA, requires a region of the 3’UTR containing G/U-rich elements (GREs) as well as the ARE/GRE-binding protein, HuR. The GRE:HuR complex promotes the partitioning of *Cebpb* transcripts to a peripheral region of the cytoplasm in tumor cells. Critically, this excludes *Cebpb* transcripts from a perinuclear region of the cytoplasm containing activated ERK1/2 (p-ERK), a C/EBPβ kinase. As a result, phosphorylation on the C/EBPβ ERK site (mouse Thr188; human Thr235), is suppressed, presumably because the newly-translated protein cannot access perinuclear ERK. C/EBPβ expressed in tumor cells nevertheless translocates to the nucleus but is maintained in a low-activity, under-phosphorylated state, contributing to senescence bypass. In addition to revealing a novel function for 3’UTRs, these findings underscore a critical role for perinuclear compartmentalization of activated ERK in *RAS*-transformed cells.

In a more recent study [6] we extended these observations by showing that another C/EBPβ kinase, CK2, exhibits RAS-induced relocation to the perinuclear cytoplasm. Moreover, the MAPK scaffold, KSR1, undergoes similar perinuclear translocation. RAS-driven re-localization of CK2 and KSR1 was observed in both NIH3T3 fibroblasts and MEFs, demonstrating that spatial reprogramming occurs regardless of whether RAS induces transformation or senescence. We have named these subcellular signaling hubs “perinuclear signaling complexes” or PSCs. Knockout or knockdown of KSR1 revealed its essential role in perinuclear targeting of ERK and CK2 in various tumor cells and in HRAS^G12V^-expressing (senescent) MEFs. In addition, KSR1 partially co-localized with CK2 and p-ERK. To identify the pathway(s) downstream of RAS that mediate PSC formation, inhibitors of CK2 and MEK/ERK kinases were tested. These agents disrupted the perinuclear localization of KSR1 as well as that of the two kinases themselves. Hence, sustained activity of ERK and CK2 is critical for cells to form PSCs and the kinases both regulate, and are components of, these signaling complexes. Moreover, there is positive cross-talk between the ERK and CK2 pathways to induce PSC formation, although the underlying mechanisms and critical phosphorylation targets remain to be elucidated. Loss of KSR1 also prevented RAS-induced phosphorylation/activation of C/EBPβ (expressed without its 3’UTR) by CK2 and ERK. Altogether, these results indicate that PSCs act as key signaling platforms required for CK2 and ERK to access specific substrates such as C/EBPβ.

Confocal imaging of ERK and CK2 in tumor cells revealed their presence in perinuclear vesicular structures, suggesting that PSCs might reside on nuclear-proximal endosomes. Supporting this idea, dynasore (a dynamin inhibitor that blocks clathrin-mediated endocytosis) disrupted PSC formation and prevented RAS-induced phosphorylation/activation of C/EBPβ expressed from a construct lacking its 3’UTR. Interestingly, overall p-ERK levels were unaffected by the drug, indicating that activation of the RAS-ERK pathway *per se* does not require endocytosis. Rather, the data indicate that targeting of ERK to the appropriate subcellular location through endosomal trafficking is crucial for its ability to phosphorylate C/EBPβ. RAS-induced phosphorylation of C/EBPβ by CK2, whose kinase activity is believed to be constitutive, was also disrupted by dynasore and the drug blocked CK2 perinuclear localization. Based on these results, we hypothesized that association with PSCs is essential for CK2 to productively interact with C/EBPβ. These observations indicate that CK2 functions as a RAS effector kinase; however, this involves spatial reprogramming (PSC formation) rather than stimulation of its intrinsic enzymatic activity.

What type(s) of endosomes are associated with PSCs? Immunostaining of various Rab GTPases, which regulate endosomal maturation and can serve as markers of specific endosomal populations, showed that Rab11 partially co-localized with CK2 and KSR1 but not p-ERK. Rab11 marks recycling endosomes, which are involved in trafficking of cargoes such as internalized receptor tyrosine kinases to the plasma membrane or other subcellular locations [7]. We found that CK2 and a fraction of KSR1 are present on Rab11^+^ recycling endosomes, whereas p-ERK and another pool of KSR1 associate with a different, as yet unidentified endosomal population.

In addition to their presence in transformed cells expressing oncogenic RAS, PSCs were also transiently induced by serum growth factors (GFs) in NIH3T3 cells. PSCs containing p-ERK, CK2 and KSR1 formed 4-6 hr post-stimulation, and rapidly decayed thereafter. These findings define a late phase of GF signaling that is not typically analyzed in signaling studies. Interestingly, CK2 and p-ERK became perinuclear with different kinetics (4 and 6 hrs, respectively), corresponding temporally with phosphorylation of C/EBPβ on its two cognate sites. C/EBPβ phosphorylation and activation of DNA binding was only observed when the protein was expressed from a construct lacking its 3’UTR, consistent with UPA preventing both RAS- and GF-induced activation of C/EBPβ in transformed and immortalized cells. Induction of C/EBPβ DNA binding was initiated at 4 hr and maintained through 6 hr, corresponding with PSC formation by CK2. These data are concordant with our observations that C/EBPβ DNA binding requires phosphorylation by CK2 but not ERK. Overall, our results support the notion that the two kinases form PSCs on distinct classes of endosomes, leading to phosphorylation of their cognate sites on substrates such as C/EBPβ with differing kinetics. The fact that PSCs transiently formed during the late phase of GF signaling appear indistinguishable from those constitutively present in tumor cells suggests that transitory perinuclear signaling drives the controlled proliferation of normal cells, while sustained PSC formation promotes oncogenesis in cancerous cells.

Notably, we have observed PSCs in all tumor cell lines examined to date, including A375 melanoma cells carrying the BRAF^V600E^ oncogene (where BRAF depletion disrupted PSC formation) as well as cancer cells such as HeLa that lack known RAS pathway mutations. Perinuclear kinases were also clearly present in Kras^G12D^- and Braf^V600E^-driven mouse lung tumors, while these structures were absent from adjacent normal tissue. Moreover, both WT and mutant BRAF proteins displayed perinuclear localization in tumor cell lines and tissues, adding another RAS pathway kinase to the list of those known to form PSCs. Collectively, these findings raise the intriguing possibility that PSCs are a universal hallmark of cancer cells and have a key role in driving tumorigenesis. We are currently exploring whether PSCs can be used as biomarkers for cancer detection and to assess therapeutic responses to drugs and disease relapse.

Future studies will seek to identify specific substrates for PSC-associated kinases in tumor cells (and in normal cells during the late phase of GF signaling) and will further dissect the pathways and proteins required for PSC formation. This research should provide valuable information about the function and regulation of these signaling centers and may reveal druggable targets for development of novel anti-cancer therapies.
